# Self-Affirmation Improves Problem-Solving under Stress

**DOI:** 10.1371/journal.pone.0062593

**Published:** 2013-05-01

**Authors:** J. David Creswell, Janine M. Dutcher, William M. P. Klein, Peter R. Harris, John M. Levine

**Affiliations:** 1 Department of Psychology, Carnegie Mellon University, Pittsburgh, Pennsylvania, United States of America; 2 Department of Psychology, University of California Los Angeles, Los Angeles, California, United States of America; 3 Division of Cancer Control and Population Sciences, NCI, Bethesda, Maryland, United States of America; 4 Department of Psychology, University of Sheffield, Sheffield, United Kingdom; 5 Department of Psychology, University of Pittsburgh, Pittsburgh, Pennsylvania, United States of America; Universidad de Granada, Spain

## Abstract

High levels of acute and chronic stress are known to impair problem-solving and creativity on a broad range of tasks. Despite this evidence, we know little about protective factors for mitigating the deleterious effects of stress on problem-solving. Building on previous research showing that self-affirmation can buffer stress, we tested whether an experimental manipulation of self-affirmation improves problem-solving performance in chronically stressed participants. Eighty undergraduates indicated their perceived chronic stress over the previous month and were randomly assigned to either a self-affirmation or control condition. They then completed 30 difficult remote associate problem-solving items under time pressure in front of an evaluator. Results showed that self-affirmation improved problem-solving performance in underperforming chronically stressed individuals. This research suggests a novel means for boosting problem-solving under stress and may have important implications for understanding how self-affirmation boosts academic achievement in school settings.

## Introduction

Acute and chronic stress have been shown to disrupt problem-solving and creativity [1]. For example, acutely stressful contexts, such as completing problem-solving tasks under negative social evaluation, have been shown to impair performance on a variety of tasks, such as anagrams and remote associate problems [2], [3]. Feeling chronically stressed produces similar performance impairments. For example, Liston and colleagues found that participants who reported high levels of stress over the previous month demonstrated impaired attention-shifting performance compared to participants who reported low levels of stress [4], [5]. Moreover, these stress-induced performance impairments were reversed when the high-stress participants completed the tasks after a one-month low stress period [4]. Although this body of research provides supportive evidence indicating that acute and chronic stressors can impair problem solving, little is currently known about stress management approaches for mitigating the effects of stress on problem solving.

An emerging body of research suggests that self-affirmation may be one such effective stress management approach. Self-affirmation theory posits that the goal of the self is to protect one’s self-image when threatened and that one way to do this is through affirmation of valued sources of self-worth [6], [7]. In order to manipulate self-affirmation, experimental studies commonly have participants rank-order personal values (e.g., politics, relations with friends/family), and then participants in the self-affirmation condition are asked to respond to questions or complete a short essay on why their #1 ranked value is important (control participants complete a similar activity about why a lower ranked value might be important to someone else) [8]. As a result, participants in the self-affirmation condition have an opportunity to affirm a valued self-domain or characteristic [6], [8]. Studies using this experimental approach have found that self-affirmation can buffer threats to the self in variety of domains [6], with several recent studies showing that self-affirmation can buffer stress responses to laboratory stressors [9], [10] and naturalistic academic stressors [11]. Collectively, this work suggests that if self-affirmation can reduce stress, it may also promote problem-solving performance under high stress conditions, although no previous studies have tested the effects of self-affirmation manipulations on actual problem-solving performance [12]–[16].

In the present study, we test whether a brief self-affirmation can buffer the negative impacts of chronic stress on problem-solving. Specifically, we used a well-known measure of problem-solving and creativity (the Remote Associates Task (RAT)) [17]–[20] to test three hypotheses. First, we tested whether chronic stress is related to poorer problem-solving performance. Second, we tested whether self-affirmation improves problem-solving. Third, we tested whether these two main effects are qualified by a chronic stress × self-affirmation interaction, such that self-affirmation will improve problem-solving in chronically stressed participants, whom are likely to have impaired problem-solving, compared to participants who are low in chronic stress.

## Methods

### Ethics Statement

This research was approved by the Carnegie Mellon University Institutional Review Board.

### Participants

Eighty students from two urban universities in Pittsburgh participated for course credit or $20. We excluded seven participants who did not follow instructions (N = 5) or who did not rate academic performance as important to them (N = 2). The sample thus consisted of 73 students (34 females; 39 males) who ranged in age from 18 to 34, with an average age of 21 (SD = 2.4). Given this broad age range and the marginally significantly association between age and overall RAT performance (*r* = −.21, *p* = .07), we controlled for age in all analyses. The ethnic composition of the sample was predominantly Caucasian (55%), followed by Asian-American (16.5%), Other (12%), African-American (9.5%), mixed-race (5.5%), and Latino/Hispanic (1.5%). The sample had similar levels of chronic stress (*M = *16.6, *SD* = 7.1, Range = 1–34) to normed US samples of individuals under 25 years of age (M = 16.8) [21]. Ethnicity (Caucasian vs. all others) and gender (male vs. female) did not moderate any of the primary study results (see Tables 1 and 2).

**Table 1:** Multiple regression analyses tested for the effects of ethnicity (coded as white vs. all other ethnic groups) on RAT problem-solving performance.

Analysis	Beta Coefficient	t-value	p-value
Main Effect	*B* = −.199	*t*(72) = −1.273	*p* = .208
Ethnicity × Affirmation	*B* = .216	*t*(72) = 1.101	*p* = .275
Ethnicity × PSS	*B* = .234	*t*(72) = 0.975	*p* = .333
Ethnicity × Affirmation× PSS	*B* = −.386	*t*(72) = −1.713	*p* = .092

Notes: PSS = Perceived Stress Scale Composite Variable; Affirmation = Self-affirmation vs. Control.

**Table 2:** Multiple regression analyses tested for the effects of gender (male vs. female) on RAT problem-solving performance.

Analysis	Beta Coefficient	t-value	p-value
Main Effect	*B* = −.012	*t*(72) = −.077	*p* = .939
Gender × Affirmation	*B* = −.132	*t*(72) = −.667	*p* = .507
Gender × PSS	*B* = −.130	*t*(72) = −.499	*p* = .620
Gender × Affirmation× PSS	*B* = .046	*t*(72) = .161	*p* = .873

Notes: PSS = Perceived Stress Scale Composite Variable; Affirmation = Self-affirmation vs. Control.

### Procedure

Participants provided written informed consent and then completed an experiment ostensibly about intelligence and performance. Participants were informed that a trained evaluator would administer the performance task. Prior to completing the RAT and while the evaluator was ostensibly preparing to administer the test, participants were asked if they would be willing to complete a questionnaire and writing activity that was being piloted for an unrelated experiment on personal values (all agreed). Participants were randomly assigned either to the self-affirmation or control condition. In both cases, they rated 11 values (i.e., art, business, friends/family) in order of personal importance. Next, they wrote about their first ranked value and why it was important to them (self-affirmation condition) or their ninth ranked value and why it might be important to others (control condition) [12]. Following the self-affirmation writing task, as a manipulation check, participants were asked to respond to two items assessing how important the value they wrote about was, using a 6-point response scale (1 = Strongly Disagree to 6 = Strongly Agree). Items were, “This value has influenced my life” and “This value is an important part of who I am” (study α = .96). Participants then completed a state mood adjective checklist assessing state positive mood (5 items: proud, content, joyful, love, and grateful; study α = .84) and state negative mood (3 items: sad, angry, scared; study α = .65) (PANAS-X; [22], [23]).

Participants’ heart rate and mean arterial pressure were measured at 2-minute intervals using an automatic sphygmomanometer and inflatable cuff on their left arm (Dinamap Carescape V100, General Electric Company, Finland) during three different periods: an eight-minute baseline period, followed by the RAT (about 9 minutes), and a five-minute recovery period. All readings in each period were averaged. Heart rate was included because it is a useful indirect marker for task engagement [24], [25], which may be affected by our self-affirmation manipulation. Mean arterial blood pressure was collected to measure cardiovascular reactivity to the laboratory challenge task.

The experimenter was blind to participant condition, and a separate RAT evaluator (also blind to condition) administered the 30-item RAT performance task. 144 RAT items have been normed for difficulty [17], and pilot testing indicated that our undergraduate sample population can solve all easy RAT items. We thus selected 30 challenging RAT items ranging in difficulty from moderately to extremely difficult (the items are available in Table S1). For each RAT item, participants saw three words on a computer screen (e.g., flake, mobile, cone) and were asked to generate a fourth word (e.g., snow) that when combined with each of the three stimulus words results in a common word pair used in everyday English language (e.g., snowflake, snow mobile, snow cone). They were given 12 seconds to provide an answer verbally. The evaluator provided veridical verbal performance feedback (incorrect, correct) after each response and recorded each response. In order to create performance pressure, the evaluator provided evaluative feedback three times during the 30 RAT trials (“I need you to try harder”).

After completing the performance task, the evaluator left the room and the experimenter re-entered and indicated that the participant was to rest quietly (5 minute recovery period). Participants then completed individual difference measures, including the 10-item Perceived Stress Scale [26] to assess perceived stress over the last month (all items were summed to form a composite index of chronic stress, study α = .87). To reduce potential confounding effects, we administered these measures at the end of the experimental session because previous studies indicate that completing individual difference measures at the beginning of an experimental session may act as an affirmation manipulation (i.e., they have carry-over effects) [27]. We had no reason to expect that the experimental task would bias participants’ responses when self-reporting their chronic stress levels over the past month, and a one-way ANCOVA indicated that the self-affirmation manipulation did not affect perceived stress over the last month (*F*(1, 72) = .95, *p* = .22, *η* ^2^ = .01). After completing individual difference measures, participants were debriefed, compensated, and excused.

### Data Analysis

All descriptive statistics, ANCOVA, and multiple regression analyses were conducted using SPSS 19.0 (IBM, Armonk, New York). All predictor variables were mean-centered prior to being entered in multiple regression equations. Our experimental manipulation of self-affirmation was dummy coded (self-affirmation = 1, control = 0). Correct responses on the RAT were summed across the 30 trials to form an overall composite RAT problem-solving performance score. As described above, age was included as a covariate in all analyses (except the preliminary chi-square analyses described below).

## Results

### Preliminary Analyses

It is possible that there may have been significant differences in how participants ranked their #1 value across study conditions, which could indicate a failure of randomization. To test whether there were differences in the selected #1 ranked value between study conditions, chi-square analyses were conducted to test for condition differences (self-affirmation vs. control, low vs. high chronic stress) on which value participants’ ranked #1 (Table 3 provides frequencies of #1 ranked values across conditions). Consistent with previous studies [28], approximately 50% of participants selected “Relations with Friends and Family” as their #1 ranked value. Importantly, there was no main effect for either self-affirmation condition (χ^2^(8) = 6.36, *p = *.61) or chronic stress level (χ^2^(8) = 6.50, *p* = .59) on the #1 ranked value. Moreover, the self-affirmation × chronic stress interaction for the #1 ranked value was not significant (χ^2^(8) = 3.03, *p* = .93). In sum, there was no evidence that self-affirmation condition or chronic stress level affected participants’ selection of their top-ranked value.

**Table 3:** #1 Ranked Value selected by participants according to self-affirmation condition and chronic stress level (as determined by median split).

	Group			
Value Chosen	Control Condition,Low Stress	Control Condition,High Stress	Affirmation Condition,Low Stress	Affirmation Condition,High Stress
Artistic Skills	0	1	0	0
Athletics	0	0	0	0
Business/Money	2	0	1	1
Creativity	1	0	3	1
Independence	2	2	2	3
Music	0	0	1	1
Politics	0	0	0	0
Relations with Friends and Family	9	12	8	8
Religious Values	3	1	2	1
Sense of Humor	1	1	1	2
Spontaneity	2	2	1	2

As expected, self-affirmation and control participants wrote about different values during the writing activity (χ^2^(10) = 33.7, p<.001; see Table 4), such that participants in the control condition wrote about a ninth-ranked value that was different from the first-ranked value in the self-affirmation condition. As shown in Table 4 and noted above, approximately half the self-affirmation condition participants wrote about relations with friends and family, whereas control condition participants wrote about a heterogeneous set of values. We had no reason to believe that chronic stress would influence choice of value. Consistent with this expectation, there was not a main effect for either chronic stress level (χ^2^(10) = 11.08, *p* = .35) nor a self-affirmation condition × stress level interaction (χ^2^(10) = 10.6, *p = *.39).

**Table 4:** Values that participants wrote about by affirmation condition and chronic stress level (as determined by median split).

	Group			
Value Written About	Control Condition,Low Stress	Control Condition,High Stress	Affirmation Condition,Low Stress	Affirmation Condition,High Stress
**Artistic Skills**	2	2	0	0
**Athletics**	2	1	0	0
**Business/Money**	3	3	1	1
**Creativity**	0	0	3	1
**Independence**	3	2	2	3
**Music**	3	1	1	1
**Politics**	0	6	0	0
**Relations with Friends** **and Family**	0	1	8	8
**Religious Values**	2	1	2	1
**Sense of Humor**	1	1	1	2
**Spontaneity**	4	1	1	2

Note that self-affirmation participants write about their #1 ranked value and control participants write about their #9 ranked value.

As a manipulation check, we compared the ratings that participants in different conditions made about their value writing activity immediately after completing the writing activity. A one-way ANCOVA confirmed that the self-affirmation group (*M* = 22.97, *SD* = 1.38) rated the value as significantly more important than did the control group (*M = *15.13, *SD* = 3.69), *F*(1, 71) = 142.6, *p*<.001, *η* ^2^ = .671, indicating success of the value-affirmation manipulation.

We also conducted an ANCOVA comparing the total number of words written in the affirmation and control essays to determine if self-affirmation participants were more engaged in the writing task and thus wrote longer essays. Although self-affirmation condition participants wrote somewhat longer essays on average (*M* = 68.79 words, *SD* = 25.9) than did control condition participants (*M* = 60.34, *SD* = 26.9), this difference was not statistically significant (*F*(1,72) = 1.63, *p* = .21). Moreover, chronic stress level was not associated with the number of words written in the self-affirmation essays (*F*(1, 72) = 1.13, *p* = .35). There was also no interaction between self-affirmation condition and chronic stress level on number of words written (*F*(1,72) = 1.30, *p* = .26). It is also worth noting that word count was not correlated with RAT problem-solving performance (*r* = .14, *p* = .23), and including word count as a covariate did not appreciably change our primary problem-solving results (word count was not further pursued as a variable of interest).

### Self-Affirmation, Stress, and Problem-Solving Performance

**Figure 1:**
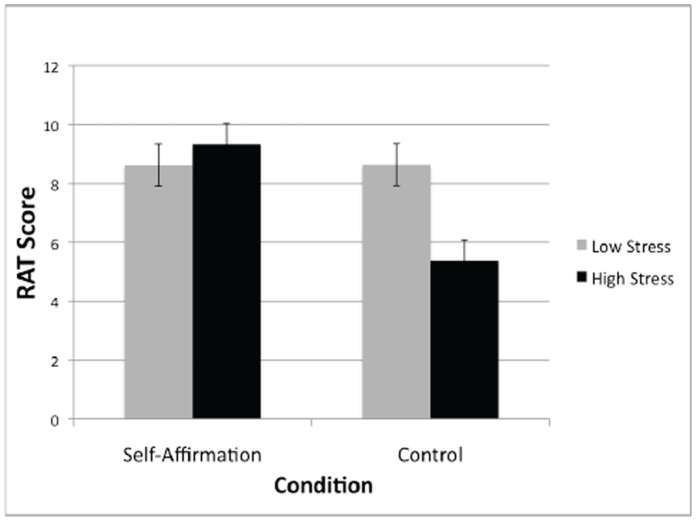
Self-affirmation effects on RAT performance in low and high chronically stressed participants. Low and high stress groups (as measured by the Perceived Stress Scale) were determined by median split for visual presentation. Error bars reflect standard errors of the mean.

To test our primary hypotheses, we conducted a multiple regression analysis with condition (self-affirmation vs. control), perceived stress over the last month, and their interaction predicting RAT score. Consistent with hypotheses, we observed a significant main effect of chronic stress on RAT performance (*β* = −.45, *t*(72) = −2.75, *p* = .008), such that participants with higher stress in the last month had lower problem-solving performance. Moreover, we observed a significant main effect for self-affirmation condition, (*β* = .31, *t*(72) = 2.88, *p* = .005), such that affirmed participants performed significantly better on the RAT task than control participants (Figure 1). Consistent with our self-affirmation stress buffering hypothesis, these main effects were qualified by a significant chronic stress × self-affirmation interaction on RAT problem-solving performance (*β* = .35, *t*(72) = 2.09, *p* = .041). As shown in Figure 1, self-affirmation (compared to the control condition) improved the RAT problem solving performance of underperforming high chronic stress individuals, but had a minimal impact on the performance of participants low in chronic stress. Moreover, as depicted in Figure 1, this stress buffering effect of self-affirmation improved the problem-solving performance of high stress individuals to a level comparable to individuals low in stress.

### Testing the Positive Affect and Task Engagement Accounts of Problem-Solving

Previous studies indicate that positive affect boosts problem-solving performance [29], [30], so we tested the possibility that the self-affirmation activity was a positive affect induction, and that positive affect engendered by self-affirmation explained the problem-solving effects. Consistent with other reports [28], we found that the self-affirmation group had higher state positive affect compared to the control group (as determined by multiple regression controlling for age: *β = .*51, *t*(69) = 4.79, *p*<.001.) We also tested negative affect using the same approach, but there was not a significant main effect for self-affirmation condition (*β* = −.12, *t*(71) = −1.06, *p* = .29) or a stress × self-affirmation interaction (*β* = −.02, *t*(71) = −.90, *p* = .37) on state negative affect. However, there was not a self-affirmation × chronic stress interaction on positive affect (*β* = .19, *t*(69) = 1.19, *p* = .24). Given that self-affirmation increased state positive affect, we conducted mediation analyses (following procedure described in [31]) testing whether state positive affect mediated the impact of self-affirmation on problem-solving. In the first step of the mediation analysis, self-affirmation increased positive affect (as described above). The second step in testing mediation consists of evaluating whether the mediating variable (positive affect) predicts the outcome variable (problem-solving performance) when entered simultaneously with the predictor variable (self-affirmation condition). This second analysis revealed that positive affect was not a significant predictor of RAT performance when it was entered as a simultaneous predictor variable with the self-affirmation condition variable (*β* = −.07, *t*(71) = −.54, *p* = .59). Thus we did not find supporting evidence for positive affect as a mediator for the self-affirmation main effect or the chronic stress × self-affirmation interaction on problem-solving performance.

**Figure 2:**
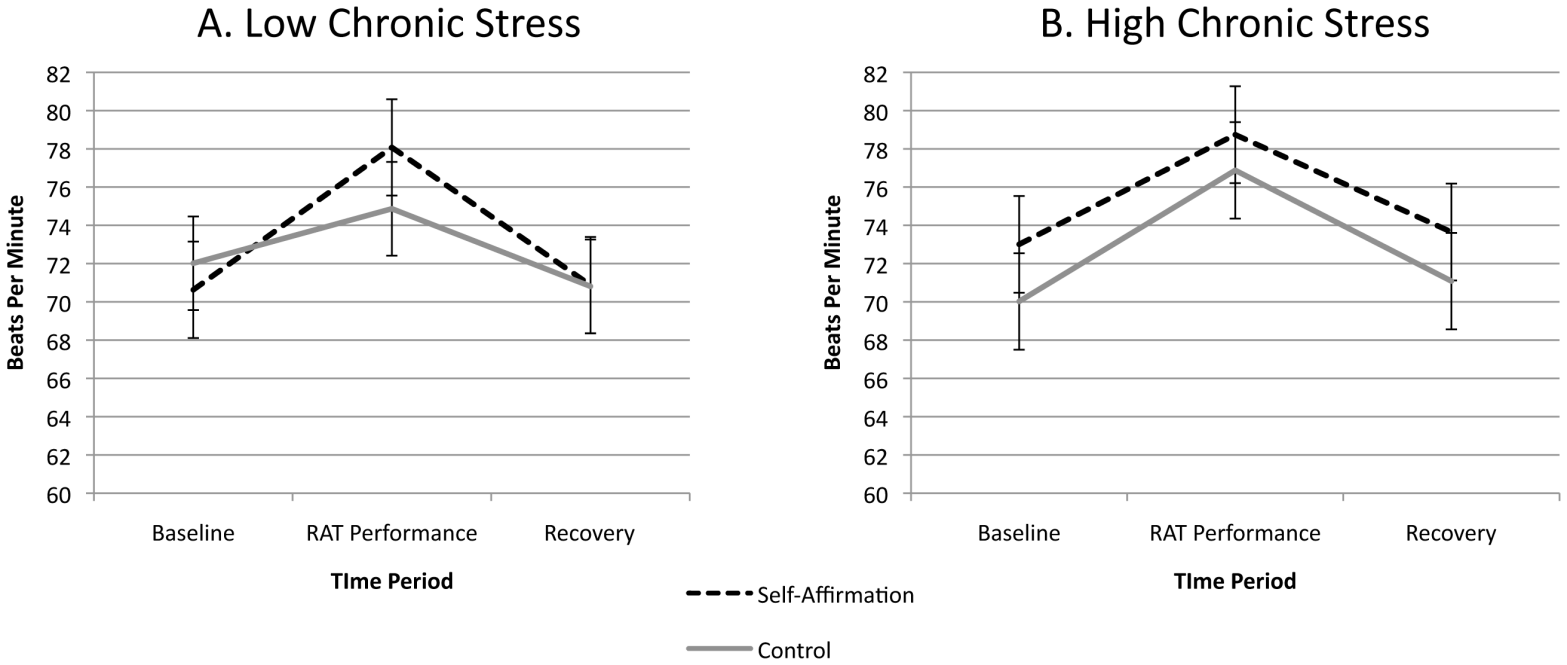
Self-affirmation effects on heart rate during the baseline, RAT performance, and recovery periods. Panel A depicts the results for participants low in chronic stress, and Panel B depicts the results for participants high in chronic stress, as determined by median split. Error bars reflect standard errors of the mean.

As noted earlier, previous research suggests that heart rate is a useful indirect marker for task engagement [24], [25]. To test whether there was differential task engagement in the self-affirmation and control conditions using this physiological measure, we conducted a repeated measures ANCOVA to assess change in heart rate over time between conditions (In order to run a parallel ANCOVA analyses as our primary analysis, the heart rate and mean arterial pressure analyses were run with the chronic stress variable entered as a two-level between subjects variable (low vs. high stress), as determined by median split). Although participants showed an overall significant heart rate increase from baseline (*M* = 68.50, *SE* = 1.03) to the RAT problem solving period (*M* = 76.44, *SE* = 1.31) (*paired-samples t*(69) = −9.26, *p* = <.001), there were no significant main effect or interactive effects of conditions on heart rate change. Specifically, we did not observe a significant main effect for self-affirmation condition (*F*(1, 67) = .36 *p* = .55, *η* ^2^ = .01) or chronic stress (*F*(1,66) = .09, *p* = .77, *η* ^2^ = .001). Notably, we also did not observe a significant self-affirmation condition × time interaction (*F*(2, 67) = .43 *p* = .65, *η* ^2^ = .01) or a condition × time × chronic stress interaction (*F*(2, 67) = 1.15 *p* = .32, *η* ^2^ = .03) (Figure 2), indicating that there were no differential effects of self-affirmation (or the self-affirmation × chronic stress interaction) on heart rate. Collectively, these findings do not provide support for a differential task engagement explanation of our performance findings. Instead, our data indicate that participants across conditions were similarly engaged in the problem-solving task.

**Figure 3:**
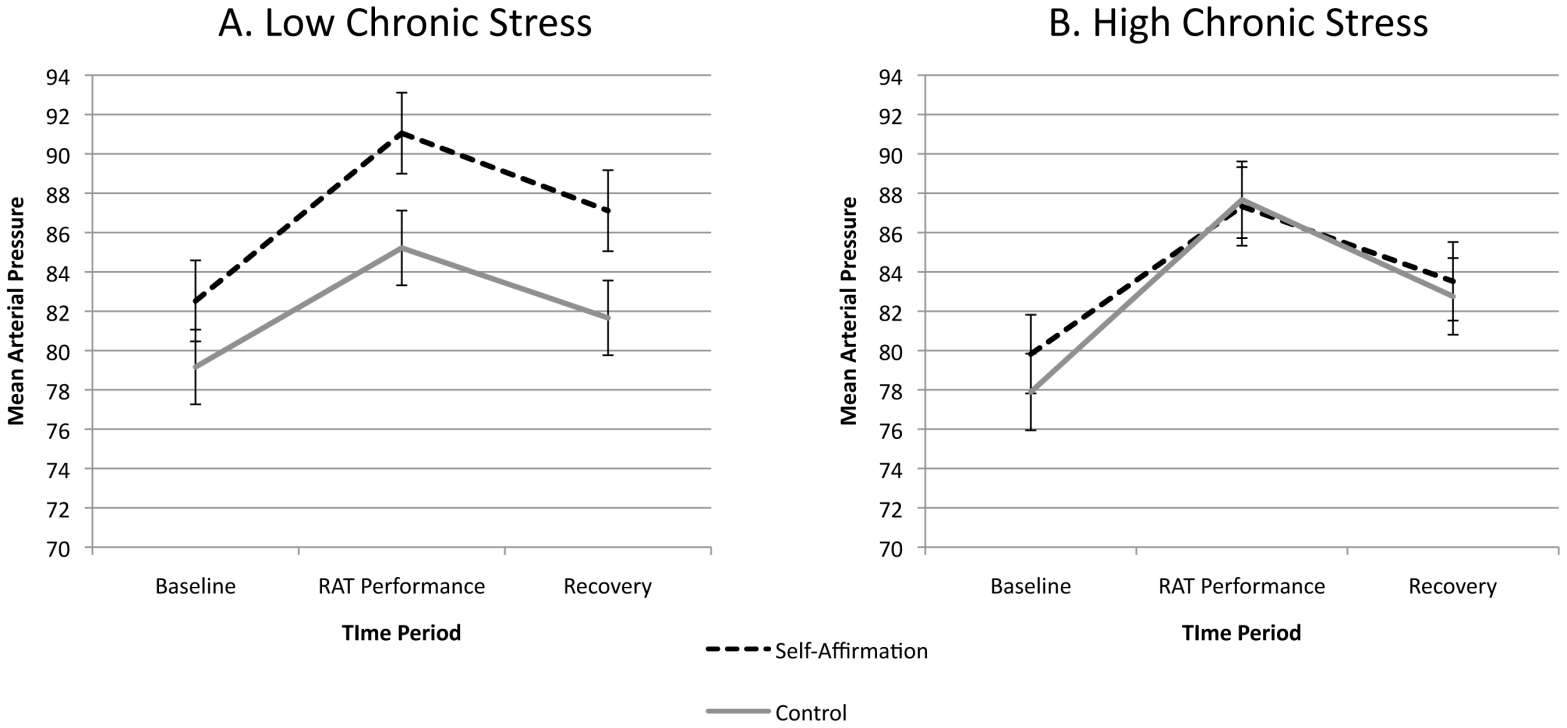
Self-affirmation effects on mean arterial pressure during the baseline, RAT performance, and recovery periods. Panel A depicts the results for participants low in chronic stress, and Panel B depicts the results for participants high in chronic stress, as determined by median split. Error bars reflect standard errors of the mean.

We also assessed the impact of our self-affirmation manipulation on mean arterial blood pressure responses during the RAT problem-solving period. Like heart rate, participants showed an overall significant mean arterial pressure increase from baseline (*M* = 79.71, *SE* = .86) to the RAT problem solving period (*M* = 89.05, *SE* = 1.08) (*paired-samples t*(69) = −12.12, *p*<.001), but we did not observe significant main effects of self-affirmation (*F*(1,67) = 2.21, *p* = .14, *η* ^2^ = .03) or chronic stress (*F*(1,66) = .32, *p* = .57, *η* ^2^ = .01). Similarly, the self-affirmation condition × time (*F*(2, 64) = .13, *p* = .88, *η* ^2^ = .004) and condition × time × chronic stress (*F*(2, 64) = 1.53 *p* = .23, *η* ^2^ = .05) interactions were not significant (Figure 3). These heart rate and mean arterial blood pressure results are in accord with our previous work showing that self-affirmation does not appreciably alter heart rate or blood pressure responses to acute stress-challenge tasks [9]. Importantly, the changes in heart rate and blood pressure reaffirm that the RAT task was stressful for participants.

## Discussion

The present study provides the first evidence that self-affirmation can protect against the deleterious effects of stress on problem-solving performance. Specifically, we show that chronically stressed individuals have impaired problem-solving performance and that self-affirmation can boost problem-solving performance under pressure. Notably, these effects were qualified by a significant chronic stress by self-affirmation interaction, such that self-affirmation improved problem-solving performance in underperforming chronically stressed individuals. These findings have important implications for self-affirmation research and educational interventions. First, although we have shown in several studies that self-affirmation can reduce acute stress experiences [9]–[11], previous research has not tested whether self-affirmation can be protective in the context of chronic (or ongoing) stressors. Moreover, until now it has been unclear whether the stress buffering benefits of self-affirmation translate into improved performance outcomes on actual problem solving tasks. Our present study suggests that a brief self-affirmation activity is sufficient to buffer the negative effects of chronic stress on task performance and can improve the ability to problem solve in a flexible manner during high stress periods [3], [32]. It is important to note that the task used in the present study (RAT) is a common measure of creativity performance and insight [18], [33], and hence our study suggests that self-affirmation may increase creativity and insight in stressed individuals [16], [34].

Second, our study suggests that self-affirmation may be effective at boosting performance in academic tasks requiring associative processing and creativity, particularly for students who experience stress on such tasks [34]. Thus, our findings identify a potential mechanism by which a self-affirmation intervention at the beginning of a school term can improve at-risk students’ academic achievement, reducing achievement disparities between African Americans and European Americans and between women and men in science [12]–[15].

Finally, two limitations of our study should be mentioned. It is possible that the stress buffering effects of self-affirmation on problem-solving performance that we obtained are specific to evaluative performance settings, since all of our participants completed difficult RAT items under time pressure in front of a critical evaluator. (We note that the problem-solving task we used produced significant cardiovascular stress reactivity (see Figures 2 & 3), comparable to other well-known psychosocial stress-challenge tasks [35].) Future studies should therefore experimentally test whether social evaluation is a necessary condition for self-affirmation problem-solving effects. Another limitation of our study is that we measured chronic stress using a self-report measure, and this measure was collected at the end of our study session (although there were no experimental (self-affirmation manipulation) effects on chronic stress scores). We elected to use this procedure given that completing individual difference measures may have carry-over effects if completed immediately prior to self-affirmation activities [27]. Future studies using other measures for assessing chronic stress (e.g., selecting chronically stressed vs. matched control groups) [4] would therefore be useful.

The present research contributes to a broader effort at understanding how stress management approaches can facilitate problem-solving performance under stress. Despite many studies showing that acute and chronic stressors can impair problem-solving [1], [2], [4], we know little about stress management and coping approaches for buffering stress during problem-solving [36]. Our work suggests that self-affirmation may be a relatively easy-to-use strategy for mitigating stress and improving problem-solving performance in evaluative settings. It will be important for future studies to consider the mechanisms linking self-affirmation with improved problem solving. We show here that our self-affirmation effects are unlikely to be explained by changes in positive affect or task engagement. The fact that we did not see any differential effects of self-affirmation on a physiological measure of task engagement (heart rate) also suggests that these effects are not driven by changes in persistence or motivation [32]. A more likely possibility, to be tested by future research, is that self-affirmation facilitates a more open and flexible attentional stance (e.g., [16]), which increases working memory availability [37], [38] for problem-solving in evaluative contexts.

### Conclusions

The present study builds on previous research showing that self-affirmation has stress protective effects in performance settings [9], [12], [13], [15], providing an initial indication that self-affirmation can buffer the effects of chronic stress on actual problem-solving in performance settings.

## Supporting Information

Table S1 **Remote Associate items used in the present study.**
(DOCX)
