# Supplementary material for: Self-Affirmation Improves Problem-Solving under Stress
Source: PLoS One. 2013 May 1;8(5):e62593. doi: 10.1371/journal.pone.0062593 (PMC3641050; doi:10.1371/journal.pone.0062593)
Supplement: Table S1 — Remote Associate items used in the present study. (DOCX) [file pone.0062593.s001.docx]

Table S1. Remote Associate items used in the study.

**Moderately Difficult**

Cream Skate Water (Ice)

Duck Fold Dollar (Bill)

Safety Cushion Point (Pin)

Worm Shelf Lend (Book)

Fish Mine Rush (Gold)

Flake Mobile Cone (Snow)

Fox Man Peep (Hole)

Pie Luck Belly (Pot)

**Difficult**

Piece Mind Dating (Game)
Lift Card Mask (Face)

Mill Tooth Dust (Saw)

Cover Arm Wear (Under)

Break Bean Cake (Coffee)

Opera Hand Dish (Soap)

Water Mine Shaker (Salt)

**Very Difficult**

Fence Card Master (Post)

Trip House Goal (Field)

Rain Stomach Test (Acid)

Office Mail Hat (Box)

Sandwich House Golf (Club)

Pine Crab Sauce (Apple)

Home Sea Bed (Sick)

Eight Skate Stick (Figure)

**Extremely Difficult**

Shadow Chart Drop (Eye)

Land Hand House (Farm)

Stick Maker Point (Match)

Forward Flush Razor (Straight)

Fight Control Machine (Gun)

Cry Front Ship (Battle)

Mail Board Lung (Black)
